# Differences in height, weight, and BMI in patients with mood disorders, schizophrenia, and other mental disorders vs. normal controls

**DOI:** 10.3389/fpsyt.2025.1710167

**Published:** 2026-01-19

**Authors:** Konstantinos N. Fountoulakis, Nikoleta Petalidou, Nikolaos K. Fountoulakis, Ioannis Nimatoudis, Pavlos N. Theodorakis

**Affiliations:** 1 3rd Department of Psychiatry, School of Medicine, Faculty of Health Sciences, Aristotle University, Thessaloniki, Greece; 2 Institute of Psychiatry, Thessaloniki, Greece; 3 Society for Neurosciences and Rehabilitation (E.N.A.), Thessaloniki, Greece, Thessaloniki, Greece; 4 Senior Advisor, Health Policy, WHO Country Office Hungary, Regional Office for Europe, Budapest, Hungary

**Keywords:** BMI, body surface areabody surface area, height, mental disorders, weight

## Abstract

**Introduction:**

The literature suggests differences in the body between the general population and patients with mental disorders. The current study aimed to search for differences between healthy controls and patients with mental disorders in terms of body height, weight, BMI, and Body surface area (BSA).

**Material and methods:**

The study sample included 788 healthy control subjects, 76 patients with unipolar depression, 36 with Bipolar disorder, 16 patients with schizoaffective disorder, 122 patients with schizophrenia, and 78 patients with other mental disorders (a mixture of severe forms of OCD, psychotic and mood disorders other than the aforementioned, as well as severe personality disorders). The diagnosis was made according to DSM-IV-TR criteria, based on a semi-structured interview using the Schedules for Clinical Assessment in Neuropsychiatry version 2.0 (SCAN v 2.0). The height and weight of all subjects were measured. The Body mass index (BMI) and the Body surface area (BSA-D according to the Dubois method) were calculated. All variables were normalized using the rank and percentile method based on the percentiles obtained from the healthy control group. The statistical analysis included a MANCOVA with the Scheffé *post hoc* test.

**Results:**

The results suggested that depressed patients are shorter than expected, while higher BMI was observed in depressed females only. No other differences among groups were identified.

**Discussion:**

The current study implies the presence of a developmental component in unipolar depression since this mental disorder is associated with a body measure (height) whose development is completed by late adolescence. An explanation could involve the presence of a biased negativistic assessment of the environment, which affects the functioning of the central melanocortin system, which controls appetite, food intake, and energy expenditure, and it is directly related to body development.

## Introduction

The first to suggest that body type is related to personality and mental disorders were Ernst Kretschmer (1888–1964) and William H. Sheldon (1899–1977). They introduced the constitutional or structural, as opposed to functional, models of psychopathology, and proposed that there is an association between specific body types, personality traits, and mental disorders, and described four body types: the pyknic, the athletic, the asthenic, and the dysplastic. They suggested that thin, aesthenic individuals are prone to introversion, schizoid tendencies, and schizophrenia, while short and round individuals are extroverted and have cyclothymic personalities and manic-depression. Sheldon added some experimental sophistication and measurement data to Kretschmer’s hypothesis. He proposed the existence of three basic morphological dimensions (endomorphy, mesomorphy, and ectomorphy) and three corresponding temperament clusters (viscerotonia, somatotonia, and cerebrotonia). Their theories are no longer of academic value, but continue to influence laypeople (1–3).

The current literature suggests that there are differences in the body between the general population and patients with mental illness, especially concerning the Body mass index (BMI), which is expected to be higher in patients, and it is attributed to medication and an unhealthy lifestyle (4–7). In terms of psychoendocrinology, the hormone ghrelin, but not leptin, is associated with BMI and depressive status (8, 9). There are also genetic studies reporting a common genetic background between early stress, high BMI, and schizophrenia, bipolar disorder, and unipolar depression (10, 11).

Height is the vertical length of the human body, and its development is completed at the latest during late adolescence or early adulthood. In healthy adults, greater height is reported to constitute both a protective factor (fewer cardiovascular and respiratory diseases) and a risk factor (more colorectal, postmenopausal breast, and ovarian cancers, and possibly pancreatic, prostate, and premenopausal breast cancers) (12–19). At the social level, there is also evidence that taller people have, on average, higher education, higher income, and higher social position (20–23). Thus, information on height and its trends may reflect a generic developmental trajectory, both in terms of genetics and of the environment during childhood, including its social, economic, and political determinants (24–26). There is limited data in the literature suggesting a relationship between body height and mental health (27, 28). There are no data on Body surface area (BSA), and the statistical analysis so far is inappropriate and prone to bias.

The current study aimed to search for differences among healthy controls and patients with unipolar depression, Bipolar disorder, schizoaffective disorder, schizophrenia, and other mental disorders in terms of body height, weight, BMI, and Body surface area (BSA).

## Materials and methods

### Study sample

The study sample included 1116 subjects, of which 788 were healthy control subjects, 76 patients with unipolar depression (recurrent major depressive disorder), 36 with Bipolar disorder, 16 patients with schizoaffective disorder, 122 patients with schizophrenia, and 78 patients with other mental disorders. The group of patients with other mental disorders included a mixture of severe forms of OCD, psychotic and mood disorders other than the aforementioned as well as severe personality disorders. These disorders were too few per individual diagnosis to constitute a special diagnostic group, and thus, they were used as a generic mental disorder reference group. There were no alcohol or substance abuse cases. The gender and age composition of the sample is shown in **Table 1**.

**Table 1:** Means and standard deviations of all variables by sex and diagnosis both in terms of raw values and percentile scores.

	N	Normal N=788		Unipolar depression N=76		Bipolar N=36		Schizoaffective N=16		Schizophrenia N=122		Other mental disorder N=78		All
		Females	Males	Females	Males	Females	Males	Females	Males	Females	Males	Females	Males	
		461	327	54	22	19	17	14	2	49	73	39	39	1116
Age (years)														
	Mean	39.33	42.04	52.11	57.27	49.58	40.76	45.43	34.00	36.02	32.73	45.31	44.13	41.16
	Strd Dev	11.18	12.28	12.70	12.58	12.70	12.35	15.19	8.49	11.47	8.31	17.66	16.58	12.84
Height (in cm)														
Raw	Mean	164.98	176.94	161.59	170.55	164.00	173.65	159.93	178.00	165.39	177.90	165.05	175.51	169.74
	Strd Dev	5.61	6.67	6.35	5.64	4.86	7.28	5.80	0.00	6.17	7.81	8.57	7.38	8.78
Percentile	Mean	51.80	51.59	35.69	24.77	46.05	38.35	27.29	56.00	54.41	55.21	53.79	44.82	49.98
	Strd Dev	27.10	27.37	29.32	21.95	27.99	31.30	25.70	0.00	30.50	29.51	30.58	30.07	28.33
Weight (kg)														
Raw	Mean	65.49	83.19	72.80	77.41	75.21	83.82	70.14	81.50	69.33	81.96	68.69	81.51	73.71
	Strd Dev	12.32	12.54	13.11	13.86	17.47	12.57	12.37	2.12	15.49	14.95	18.44	14.61	15.42
Percentile	Mean	46.85	51.46	63.35	37.14	64.58	55.35	58.57	51.00	55.04	47.42	54.03	47.67	50.07
	Strd Dev	27.13	27.23	28.25	28.79	32.92	30.36	28.92	7.07	30.71	31.30	32.95	32.26	28.54
BMI														
Raw	Mean	24.09	26.54	27.98	26.56	27.84	27.80	27.43	25.73	25.36	25.91	25.10	26.36	25.50
	Strd Dev	4.50	3.55	5.29	4.13	5.72	4.02	4.60	0.67	5.51	4.61	6.06	3.87	4.57
Percentile	Mean	46.11	50.67	67.94	49.55	66.58	60.12	67.36	46.00	53.98	44.92	53.41	50.00	50.06
	Strd Dev	26.95	26.75	27.48	33.23	31.30	31.49	25.98	7.07	30.28	32.15	30.95	32.13	28.50
BSA-D														
Raw	Mean	1.72	2.00	1.77	1.89	1.81	1.98	1.73	2.00	1.76	1.99	1.74	1.97	1.84
	Strd Dev	0.15	0.16	0.15	0.17	0.21	0.16	0.15	0.02	0.18	0.18	0.24	0.19	0.21
Percentile	Mean	47.83	58.69	58.78	38.41	63.05	56.76	50.79	60.50	55.43	56.58	54.08	53.44	53.13
	Strd Dev	27.85	25.59	29.64	24.30	34.41	29.32	31.26	4.95	32.09	28.67	35.25	29.65	28.40

All control subjects and patients gave informed consent, and the protocol received approval from the University’s Ethics Committee.

### Clinical diagnosis

The diagnosis was made according to DSM-IV-TR criteria on the basis of a semi-structured interview using the Schedules for Clinical Assessment in Neuropsychiatry version 2.0 (SCAN v 2.0) (29). The SCAN is one of the most reliable and valid structured interviews endorsed by the World Health Organization.

Data on treatment or lifestyle were not registered. Specifically for medication, the main reason was that it is challenging to quantify exposure to specific medication when they are administered simultaneously in heterogeneous combinations of agents and dosages. Additionally, there is no clear class effect concerning weight gain and metabolic parameters, which are also dose-dependent to a significant extent. Thus, the design of such a study seemed methodologically impossible (the lack of a class effect was the main reason). The same was true for lifestyle factors, including dietary habits, calorie intake, smoking etc.

### Somatometric measurement

The height and weight of all control subjects and patients were measured. The subjects’ weight was measured with standardized weighing machines to ensure reliable measurements and comparability across machines. BMI was calculated as the ratio of weight in kilograms to the square of height in meters. The Body surface area (BSA) was calculated in square meters according to the Dubois method (BSA-D) (30, 31) with the formula BSA-D = 0.007184 * Height ^0.725^ * Weight ^0.425^ (height in cm, weight in kg). The means and standard deviations of the subgroups of the study sample are shown in **Table 1**.

### Statistical analysis.

The first step in the analysis was to transform the data (shown as means and standard deviations in **Table 1**) to percentile scores separately for males and females on the basis of the values obtained from the healthy controls group alone. The Rank and Percentile method was used. In this way all subjects obtained a normalized value that took into consideration also the sex of the subject and on the basis of the curve of healthy controls alone.

The statistical analysis of percentiles included Multiple Analysis of Covariance (MANCOVA) with diagnosis and gender as grouping variables, age as covariate and height, weight, BMI and BSA as dependent variables (variables transformed to percentile scores). An interaction of sex with diagnosis was also allowed. The Scheffé was used as *post-hoc* test analysis (32). The basic assumptions for the use of MANCOVA were met after transformation (normality, independence of observation, homogeneity of variances and homogeneity of covariances). The size of the smallest group permitted for two covariates.

The use of sex as a covariate in an analysis where the variables were normalized with sex already taken into consideration, could identify any effect of sex beyond the normally expected on body measurement.

## Results

The MANCOVA results suggested an effect of diagnosis (p<0.001), sex (p<0.001), and the interaction between diagnosis and sex (p=0.008), but not for age. The detailed results are shown in **Table 2**.

**Table 2:** MANCOVA results.

	Wilks	F	Effect df	Error df	p
diagnosis	0.950	2.86	20	3653	<0.001
sex	0.917	24.95	4	1101	<0.001
diagnosis*sex	0.966	1.92	20	3653	0.008

The Scheffé test *post-hoc* tests revealed significant differences in terms of height percentile between males with unipolar depression and female controls (p=0.048), and males with schizophrenia (p=0.043) with patients with unipolar depression being shorter (in terms of percentile scores) in comparison to the other groups. In terms of weight percentile there were no significant differences among groups. In terms of BMI percentile, female patients with unipolar depression had significantly higher BMI in comparison to female controls (p=0.002) and males with schizophrenia (p=0.034). In terms of BSA-D percentile, only control males differed from control females (p=0.002).

The distribution of the dependent variables within each diagnostic group showed a single peak for all variables in all groups.

## Discussion

The current study reported that patients with depression are shorter than expected, while higher BMI (in terms of percentile) was observed in depressed females only. No other differences among groups were identified. The finding that control males differed from control females in BSA-D percentile is probably an artifact of a skewed distribution. The results point to a continuum among groups concerning height and BMI (in percentiles), with male patients with schizoaffective disorder being taller and heavier, controls somewhere in the middle, and male patients with depression being shorter (in terms of percentile scores; **Table 1**). The histograms suggest the distributions of these variables do not manifest multiple peaks. The results of the current study come from a multivariable analysis that utilized variables transformed into percentiles. This means that the analysis, based on percentiles rather than raw measurements, revealed the true significance of height and BMI independently of sex and the other variables that could have acted as confounders. However, lifestyle and medication were not taken into account. We consider that the above results, which to some extent differ from previous literature, are the consequence of the very distinct way of data transformation and analysis. This method eradicated much of the bias inherent in previous papers. Taken together, the current study suggests that patients with depression manifest a somatic type different from healthy control subjects as well as from other patients with mental disorders. This type is characterized by shorter-than-expected stature and a more robust figure, probably resembling the ‘pyknic type’ of Ernst Kretschmer (1888–1964) and William H. Sheldon (1899–1977). As mentioned in the introduction, these authors proposed that short and round individuals are extroverted and have cyclothymic personalities and manic-depression (1–3).

Concerning mental health, reports are suggesting a positive relationship between height and neurocognition and especially executive functions (33). This seems to be more pronounced in males (34). However, the reports concerning the relationship of body height with mood are conflicting, with some studies suggesting a relationship between shorter stature and depression (27, 28). In contrast, others report no relationship of height with mood or suicidality (4, 33, 35, 36). It is not clear whether this potential relationship could be a consequence of treatment with antidepressants during childhood or a core feature of early-onset depression, also in the frame of a psychotic disorder (28).

BMI is much better studied both in the general population as well as in patients, in both a developmental/genetic approach as well as a consequence of mental disorder and its treatment.

In the general population, higher BMI is related to somatic/vegetative symptoms and cognitive symptoms of depression, but not with mood per se (37). However, there is data suggesting this relates to atypical depressive symptoms (38). Also, patients with morbid obesity are reported to have higher levels of depression (39). In young adolescents, the relationship between body size and depressive symptoms is curvilinear and is moderated by sex. Heavier-than-average and underweight girls, as well as boys with obesity, were reported to have the highest depression scores (6). In young adults, the relationship between BMI and eating behaviors is complex and differs between sexes, likely also due to younger age and higher metabolism (40, 41). Ghrelin but not leptin levels were associated with BMI and depressive status (8, 9). This obesity-depression relationship seems to be stronger in countries with poor socioeconomic conditions (7), and it could be related with the finding that exposure to trauma during adolescence is associated with greater waist circumference during the early phase of mental disorder (42). Our results do not support the presence of a curvilinear relationship or the presence of multiple peaks in the distribution of any studied variable.

At the genetic level, an extensive polygenic overlap between BMI and schizophrenia, bipolar disorder, and unipolar depression has been reported (10). Interestingly, higher BMI polygenic scores correlate with the presence of higher early life stress, which in turn predicted higher current depressive symptoms (11). More complex developmental models suggest that both too low and too high BMI during late adolescence correlate with the development of mood disorders throughout all the adult life (36, 43).

In adult patients with depression, cross-sectionally, there is a strong correlation between BMI and depressive symptoms in both sexes (4, 5), and this relationship seems to be stronger in females (27, 43), which is in accord with our findings. Some authors again propose that higher BMI is related to the atypical form of depression (44). Again, as seen in the general population, also in patients, very low BMI also correlates with depressive symptoms (45, 46), and this is not in accord with our findings. Another strong correlation concerned the waist-to-hip ratio and depressive symptoms (47). The biochemical picture is more complex but suggests that serum LDL-C, and triglycerides are related to depressive symptoms (46). The data on adiponectin levels are conflicting (48, 49).

Concerning other mental disorders, there is no relationship between BMI and positive symptoms in unmedicated subjects in high risk for psychosis (50). However, high BMI is common among patients with psychosis (51) as is obesity and diabetes (52), and all the laboratory predictors of metabolic syndrome (53, 54). This is not in accord with the findings of the current study, which, however, agrees with a previous study of our group, which reported negative results concerning the presence of metabolic syndrome in a population of psychotic patients (55).

Overall, the results of the current study imply the presence of a developmental component in unipolar depression since this mental disorder is associated with a body measure (height) whose development is completed by late adolescence. The question of the mediating mechanisms is difficult to answer, and only speculation can be made at present. We know that the length of the human body is determined by the interplay of many genetic and environmental variables and manifests significant differences across countries, populations, and historical times (12, 56, 57). A working hypothesis could implicate the hypothalamus and, more specifically, the central melanocortin system, which acts through melanocortin 3 and 4 receptors (MC3R and MC4R) to control appetite, food intake, and energy expenditure, and is directly related to body development (58, 59). Whether mutations of the genes controlling the structure of these receptors (57, 60) or some kind of impaired input or regulation of their function could be held responsible, should be the focus of targeted research (61).

The melanocortin system seems to act as recipient of inputs reflecting nutrient sensing, which probably corresponds to a non-conscious assessment of the available resources in the environment. This impaired input could be considered to constitute a somatic analogue of the negativist way that dominates thinking and general attitude in depressed individuals concerning the self, the environment, and the future. If such a ‘negativistic’ assessment of environmental resources exists, and acts in combination with a negativistic assessment of future availability to influence the melanocortin system, this could theoretically lead to shorter stature.

If this would prove to be correct, then it could reflect a more generalized trait tendency of the human organism towards a non-favorable interpretation of the environment. This is not restricted to mood and thought content, but also it concerns the total bodily function. This tendency leads to self-restricting and under-achieving of the total body functioning, maybe years before the actual mental disorder of depression is manifested. It could also fit well into the riddle of the relationship among early stress, later development of depression, and somatic conditions. Such an explanation could also account for these patients’ poor health and shortened life expectancy.

## Strengths and limitations

The most important strength of the current study is the large study sample, the use of multiple somatometric indices and the utilization of a sophisticated method of normalization and analysis.

The most important limitation is the lack of lifestyle and medication data, which are, in any case, very difficult to include in such an observational research project.

## Author's note

All authors confirm that the following manuscript is a transparent and honest account of the reported research. This research is related to a previous study by the same leading author titled ‘Psychophysiological Differences in Patients with/without Schizophrenia: A Comparative Study’ by Theofilidis A and Fountoulakis K., published in the Int J Clinical Biostatistics and Biometrics. The previous study was performed on the same sample but included only BMI, while the current one also includes The Body surface area (BSA). The analysis also differs, since the earlier included MANOVA, the current one includes MANCOVA (with covariance, thus it corrects for confounding factors). These differences resulted in different results.

## Data Availability

The original contributions presented in the study are included in the article/supplementary material. Further inquiries can be directed to the corresponding author.
